# Detecting virus-specific effects on post-infection temporal gene expression

**DOI:** 10.1186/s12859-019-2653-4

**Published:** 2019-03-29

**Authors:** Quan Chen, Jun Zhu

**Affiliations:** 1 Department of Genetics and Genomic Sciences, Icahn School of Medicine at Mount Sinai, New York, 10029 NY USA; 2 Icahn Institute for Genomics and Multiscale Biology, Icahn School of Medicine at Mount Sinai, New York, 10029 NY USA; 3 Sema4, a Mount Sinai venture, Stamford, 06902 CT USA

**Keywords:** Temporal association, Dynamic response, Flu virus

## Abstract

**Background:**

Different types of viruses have different envelope proteins, and may have their shared or distinctive host-virus interactions which result in various post-infection effects in humans and animals. These effects often do not appear at once but take time to unfold. To characterize the virus-specific effects, we applied a Multivariate Polynomial Time-dependent Genetic Association (MPTGA) method, previously proposed for detecting differences in temporal gene expression traits, to test for the differences in mouse lung transcriptome response to infection of different subtypes of influenza A viruses.

**Results:**

We compared two methods: the Multivariate Polynomial Time-dependent Genetic Association (MPTGA) method, and the conventional modified t-test, to study the virus-specific effects on mouse lung gene expression. Both methods found H3N2 to be the most different virus among the three viruses tested, with the largest number of genes with H3N2-specific effects. However, the MPTGA method demonstrated much higher power of detection, and the detected genes with virus-specific effects showed better biological relevance.

**Conclusions:**

Transcriptome response to virus infection is dynamic. MPTGA which leverages temporal gene expression traits showed increased power in detecting biologically relevant virus-specific effects comparing with conventional t-test method.

## Background

Influenza viruses have various types and subtypes that differ in their morbidity, virulence and pathogenicity. Influenza A virus is the most virulent type and can be further divided into several subtypes [1]. These subtypes also include different strains which may evolve over time and vary in their manifestations: zoonotic, pandemic and seasonal [2]. The highly pathogenic avian influenza H5N1 virus, for example, is a zoonotic influenza virus that is transmitted from poultry to humans. The H5N1 virus strain A/Vietnam/1203/04 was the most pathogenic strain from the 2004 outbreaks of H5N1 influenza viruses [3]. The pandemic H1N1/09 virus strain, on the other hand, has led to the swine-origin H1N1 flu pandemic in 2009. The seasonal H3N2 vaccine seed strain A/Wyoming/03/2003 represents H3N2 viruses isolated during the last three seasons [4].

Previous studies [5] have shown in time course experiments that seasonal H3N2, pandemic H1N1, and zoonotic H5N1 viruses differ strongly in the spatial and temporal dynamics of infection and associated lesions in the ferret respiratory tract. Therefore, to characterize and to differentiate the infection processes with the diverse categories and subtypes of influenza viruses, it is important to acknowledge its highly dynamic nature.

In this paper, we investigated temporal mouse lung gene expression traits in response to infection with influenza A/California/04/09 (H1N1), A/Wyoming/03/03 (H3N2), and A/Vietnam/1203/04 (H5N1) HALo virus (GSE98527) and aimed to identify the virus-specific effects. To compare temporal gene expression traits, we proposed a multivariate polynomial temporal genetic association (MPTGA) method [6] previously in the context of genetic association with temporal gene expression. We further extended this method to a more general setting that compares the temporal gene expression trait under different conditions, which applies to the specific aim in this study—to detect the differences in the way each virus affects temporal gene expression traits over time. We also compared the MPTGA method with the conventional modified t-test to detect differential gene expression post infection of different viruses, and demonstrated significant increase in the power and specificity of our temporal method.

## Results

### Mouse lung dataset

To characterize the cellular transcriptome response of mouse lung to infection of H1N1, H3N2, and H5N1 influenza virus, six to eight week-old female C57BL/6 mice were infected with influenza A/California/04/09 (H1N1), A/Wyoming/03/03 (H3N2), and A/Vietnam/ 1203/04 (H5N1) HALo virus in 3 experimental groups respectively. The Influenza A/Vietnam/ 1203/04 (H5N1) HALo mutant virus is an attenuated H5N1 virus generated from wild-type Influenza A/Vietnam/1203/04 (H5N1) virus [7]. Lungs were collected from each experimental group with 3 mice infected with the same virus at 12h, 1d, 2d, 3d and 4d post infection. The cellular transcriptome at each time point was profiled by mRNA-Seq technology (GSE98527).

### Detecting virus-specific effects on post-infection temporal expression

We applied MPTGA to all 24,421 genes profiled in the mouse lung dataset, testing for differential temporal post-infection gene expression between infection with each virus and the other two. In addition, we applied the moderated t-test using an empirical Bayes method implemented in the R package *Limma* [8] to test for differential expression based on the last time point.

**Fig. 1:**
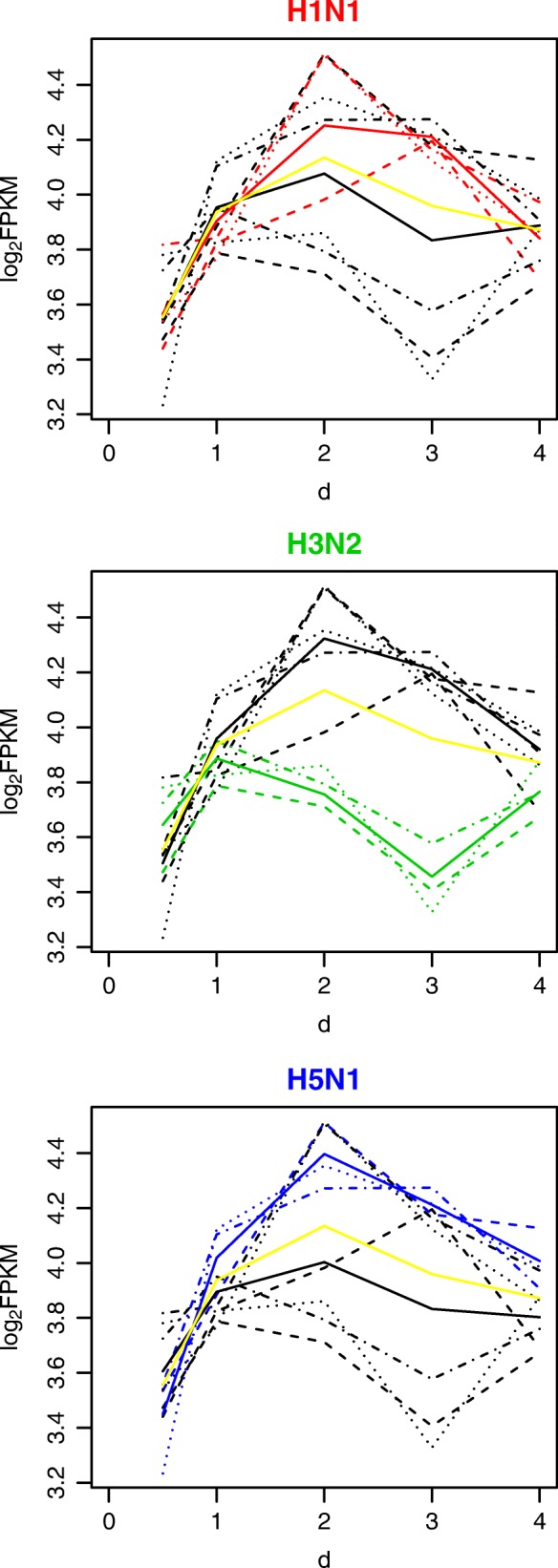
Gene expression trajectories of the Mill2 gene in mouse lung. Each dashed line represents the post-infection gene expression trajectory for a sample. Dashed lines with the same line type but different colors correspond to the same sample infected with different viruses. Red, green, blue dashed lines each corresponds to the gene expression trajectory with H1N1, H3N2, H5N1 infection, respectively. Black dashed lines correspond to the gene expression trajectory with the other 2 viruses infection. Solid lines are the fitted curves. Red, green, blue solid lines each corresponds to the fitted curve for the temporal gene expression trajectories with H1N1, H3N2, H5N1 infection, respectively. Black solid lines correspond to the fitted curve for the temporal gene expression trajectories with other 2 viruses infection. Yellow solid lines correspond to the fitted curves using the reduced model taking all samples together

**Fig. 2:**
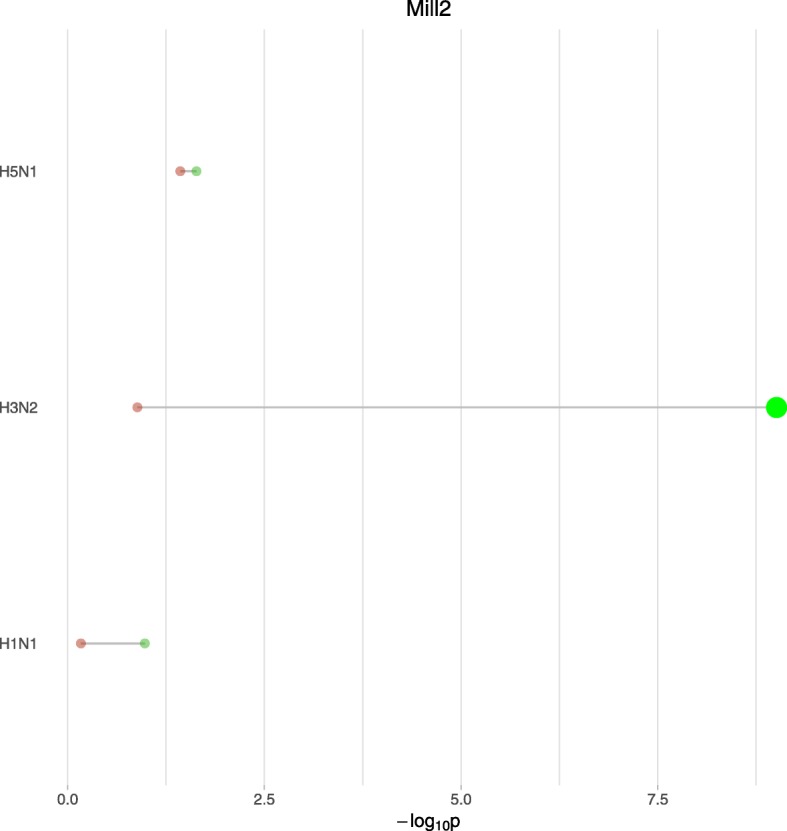
MPTGA v.s. standard differential expression test on Mill2 gene. The *p*-values of differential temporal gene expression tests on Mill2 post-infection trajectories using MPTGA and the *p*-values of standard differential gene expression tests on Mill2 based on the last time point. Green represents MTPGA, red represents standard differential expression test. Significant *p*-values are highlighted

Genes have different temporal trajectories in response to different subtypes of viruses. For example, Fig. 1 shows expression of Mill2 (MHC I like leukocyte 2), an mouse ortholog for the MICB gene in human. Mill2 is highly expressed in mouse lung according to Mouse ENCODE transcriptome data [9]. It involves in immunoregulatory interactions between a lymphoid and a non-lymphoid cell and innate immune system. Its related GO processes include immune response and immune response-activating cell surface receptor signaling pathway. Figures 1 and 2 illustrate the performance of different methods in detecting virus-specific effects on post-infection temporal expression of Mill2. Previous studies [10–14] have investigated the immune-related functions of Mill2 (or its orthologs) and have characterized the underlying mechanisms. MICB activates the cytolytic response of natural killer (NK) cells and CD8 T-cells. As is shown in Fig. 1 with H3N2 infection, Mill2 expression trajectories demonstrate an evidently distinct pattern from the trajectories post-infection with the other two viruses, consistent with previous observations that H3N2 is less effective in induction of immune response in lung [15]. However, at the last time point, all trajectories return to similar levels of gene expression values, which suggests that the standard differential expression test based on the last time point was not able to detect the difference, whereas the MPTGA method was able to detect the subtype specific effect of post-infection temporal expression of Mill2, as shown in Fig. 2.

**Fig. 3:**
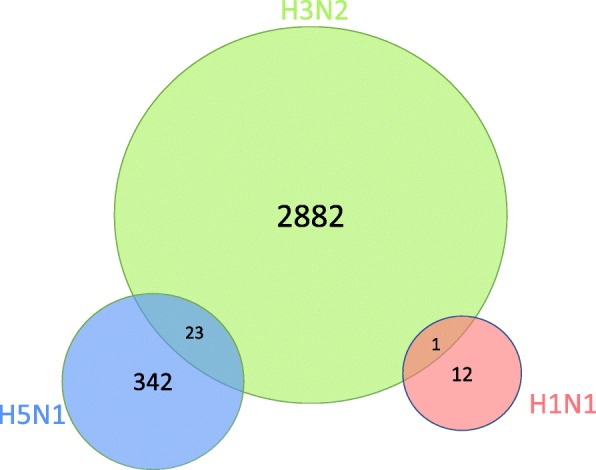
Venn diagram of MPTGA detected genes with virus-specific effects. Venn diagram of the identified genes with significant differential post-infection temporal gene expression specific to each virus, detected by MPTGA. Bonferroni correction was applied to correct for multiple testing

**Fig. 4:**
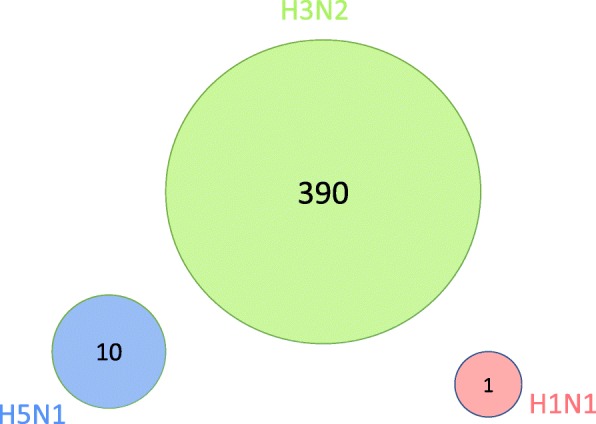
Venn diagram of *Limma* detected genes with virus-specific effects. Venn diagram of the identified genes with significant differential post-infection gene expression specific to each virus, detected by *Limma* based on the last time point. Bonferroni correction was applied to correct for multiple testing

Figure 3 provides a summary of the genes identified by MPTGA with differential post-infection temporal gene expression specific to each virus. Figure 4 provides a summary of the genes identified by the conventional differential gene expression test implemented by the R package *Limma* [8] as a moderated t-test based on the last time point. MPTGA identified more temporal differential expression genes than the conventional test, with 3260 genes showing differential temporal gene expression specific to at least one virus, while only 401 genes were identified by the conventional method. In addition, the conventional test results in three disjoint sets of genes with differential gene expression specific to each virus, suggesting that the test is only able to distinguish the post-infection effect on the gene expression between at most one of the viruses and the others. On the contrary, MPTGA identified 24 genes with differential temporal expression specific to multiple viruses. These results demonstrate significant superiority of the MPTGA method over the conventional method in both the power of detection and the specificity of virus-specific effects.

In spite of the differences, both MPTGA and the conventional test resulted in the largest number of differentially expressed genes specific to H3N2. The same observation also holds for the genes uniquely specific to each virus, with H3N2 having the largest number of genes that are only found to take on a post-infection effect specific to H3N2 but not to H5N1 nor H1N1. On the contrary, H1N1 appears to be the least specific virus, with the smallest number of a temporal effect specific to H1N1.

### Functional enrichment

**Fig. 5:**
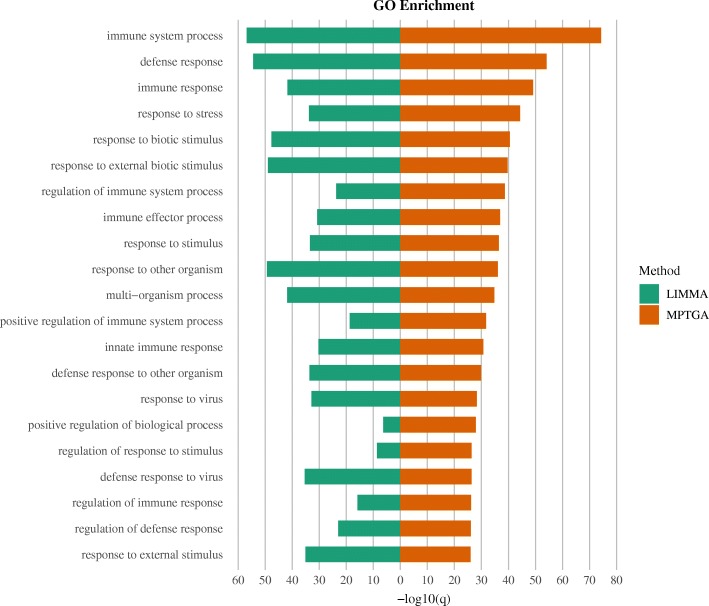
GO enrichment of H3N2-specific genes. Comparison of GO enrichment from the 2882 genes showing H3N2-specific effects only (without being specific to H1N1 or H5N1) identified by MPTGA and from the 390 genes showing H3N2-specific effects only identified by *Limma*. GO categories are ranked by the significance level obtained from the MPTGA identified 2882 genes

**Fig. 6:**
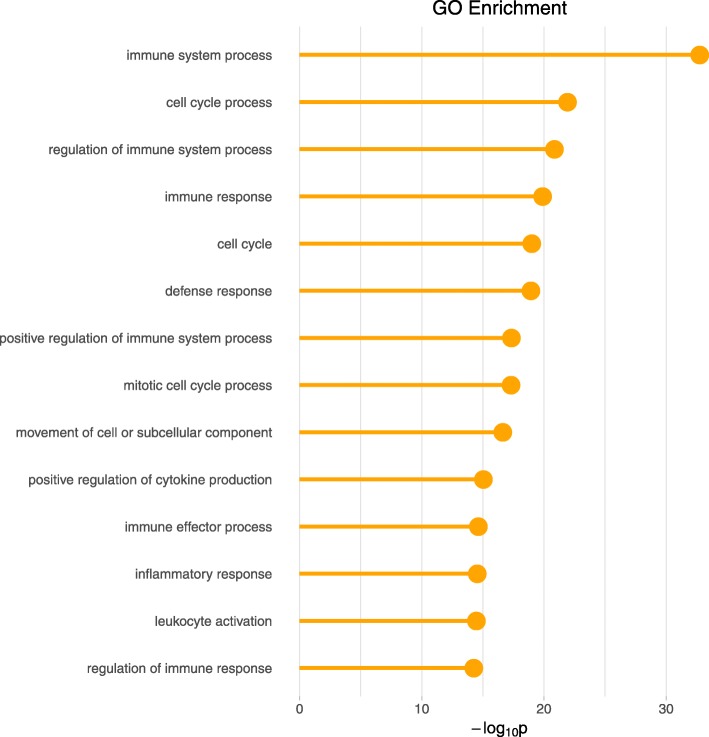
GO enrichment of H3N2-specific genes detected by MPTGA only. GO enrichment from the 2501 genes showing H3N2-specific effects only (without being specific to H1N1 or H5N1) identified by MPTGA but not identified by *Limma*. GO categories are ranked by the significance level. General GO terms with more than 1500 genes associated are not displayed here

We looked into the functional enrichment in the identified differentially expressed genes specific to H3N2 only (without being specific to H1N1 or H5N1), as H3N2 was shown to be the virus with the most specific post-infection effects by both the MPTGA and the conventional method. The MPTGA method identified 2882 genes of only H3N2-specific effects, whereas the conventional method identified 390 genes of only H3N2-specific effects. Among them, 381 (98%) genes were in the common between the two. We compare the significance level of the top enriched GO categories for the 2882 genes identified by MPTGA with the significance level of these GO categories for the 390 genes identified by the conventional method in Fig. 5. The majority of the top enriched GO terms from the MPTGA identified 2882 genes of only H3N2-specific effects were related to immune or defense mechanisms. Comparing with *Limma*, the MPTGA method shows a consistently stronger enrichment in the immune related categories. We also look into the 9 genes identified by *Limma* but not by MPTGA and find no function enrichment in any immune or defense related GO categories. Next, we show the GO enrichment of the 2501 genes among the set of 2882 genes identified by MPTGA but not by *Limma* in Fig. 6. Most of the top enriched GO terms are related to immune/defense mechanisms or cell cycle regulation.

Previous studies [16–22] have compared the pathogenesis of different viruses in human cases and animal models. The H5N1 and H1N1 subtype viruses are more pathogenic and have a larger effect on lower respiratory tract comparing the H3N2 subtype virus. The pandemic 2009 H1N1 virus is shown to have similar pathogenesis to other influenza A viruses with high virulence such as the H5N1 viruses, with extension of its inflammation process of the larger airways into the alveoli causing diffuse alveolar damage, while the less virulent seasonal H3N2 virus is found to have less extension of its inflammatory process to alveoli. The difference in the morbidity and pathogenesis observed in these viruses is consistent with what we find in comparing the effect of each of the viruses on the temporal gene expression after infection, and finding the most number of genes showing specific effects unique to H3N2. Our results showed that H3N2 specific effects in lung were related to immune response and cell cycle regulation, indicating that the MPTGA method did not only detect more genes with virus-specific effects, but also revealed significant biological pathways underlying virus-specific effects.

It has been shown that innate lymphoid cells (ILCs) accumulated in lung after virus infection to promote lung tissue homeostasis [23]. Monticelli et al. [23] reports a ILC enriched signature consisting of 121 genes. We compared the signature with our virus specific genes and identified 41, 1, and 0 genes in the overlap with H3N2, H1N1, and H5N1 specific genes, respectively. The significant overlap with H3N2 specific genes (Fisher’s Exact test *p*-value = 5.0e-10) suggests a potential mechanism explaining why H3N2 is least pathogenic to lung tissues.

## Discussion

Virus infection in humans and animals is a dynamic process, with wildly different virulence and pathology specific to types or subtypes of viruses. For health-care professionals, it is essential to understand the virus-specific effects over a temporal dimension in order to accurately identify the virus and to proceed with appropriate treatment strategies.

In this paper, we studied the temporal gene expression response in mouse lung to infection with a seasonal H3N2 virus strain, a pandemic H1N1 virus strain, and a zoonotic H5N1 virus strain. We aimed to identify the virus-specific effects on the post-infection gene expression. For this purpose, we compared two methods, a Multivariate Polynomial Time-dependent Genetic Association (MPTGA) method proposed for temporal gene expression traits, and the conventional modified t-test for differential gene expression detection. Both methods showed that the seasonal H3N2 virus was the most different among the three tested, showing distinct response in the expression of the largest number of genes. The MPTGA method identified significantly more genes with virus-specific effects for each of the three viruses comparing with the modified t-test. We looked into specific examples and observed that the effects of the viruses infection varied over time, which explained why the modified t-test failed to capture the post-infection effects for many of the genes based on a single static time point. Moreover, by comparing the genes with virus-specific effects identified by the two methods, we showed that the MPTGA method identified more genes involved in immune response and cell cycle regulation pathways, consistent with previous studies comparing patho-physiological effects of these virus subtypes. Nevertheless, what virus-host interactions drive the virus specific effects can not be derived from this study. Further studies are needed to elucidate causal regulations leading to the virus specific effects.

**Fig. 7:**
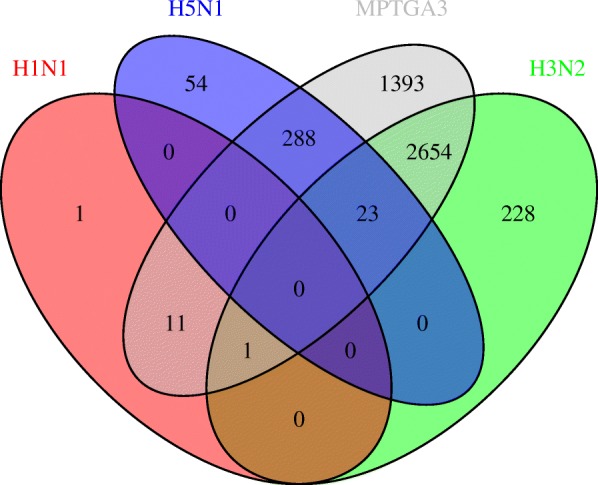
Venn diagram of MPTGA2 and MPTGA3 detected genes with virus-specific effects. Venn diagram of the identified genes with significant differential post-infection gene expression, detected by MPTGA2 and MPTGA3. Bonferroni correction was applied to correct for multiple testing

We also generalized the MPTGA model based on two genotypes/groups to three or more genotypes/groups (the MPTGA models based on 2 or 3 genotypes/groups are noted as MPTGA2 and MPTGA3, respectively). We applied MPTGA3 to the mouse lung data set and compared results based on MPTGA2 and MPTGA3 (Fig. 7). MPTGA3 identified more genes differentially regulated among 3 subtypes of viruses. There were also genes identified by MPTGA2 but not by MPTGA3, suggesting the model selection should be based on underlying data distributions. For results based on MPTGA2, it is easier to interpret common of viruses and unique to each specific virus. However, it is not easy to biologically interpret the results based on MPTGA3. Thus, we focused on the results based on MPTGA2 in the “Results” section.

**Fig. 8:**
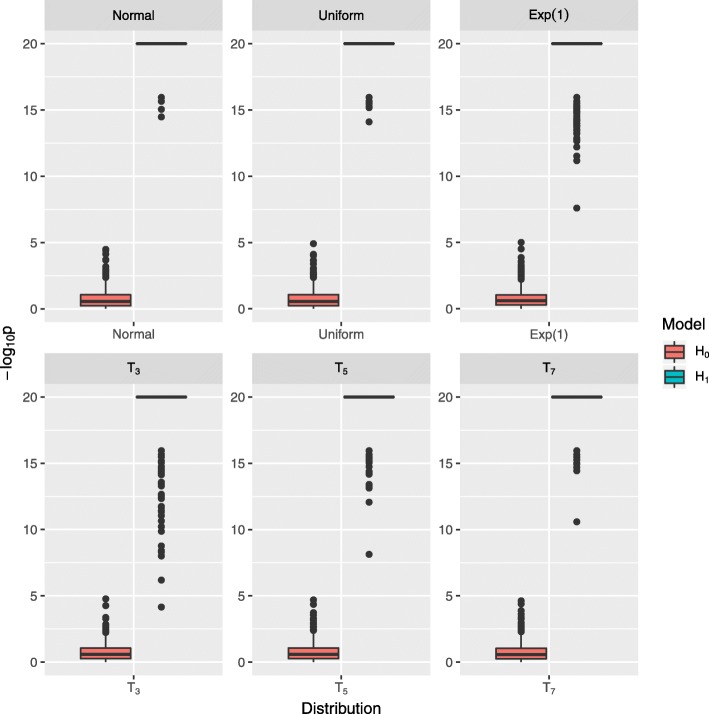
Evaluation of MPTGA performance robustness to deviations from a normal distrubtion. Summary of the MPTGA test performance when the underlying assumption of normal distribution is met or when the expression traits follow different distributions (normal, uniform, exponential, Student’s t with 3,5, and 7 degrees)

In the MPTGA model, we assumed expression levels of a gene followed a group mean trajectory with variance at each time following a normal distribution. To assess the performance of MPTGA in the cases where variances deviate from a normal distribution, we simulated data sets based on the empirical patterns identified in the mouse lung data and added residuals sampling from different distributions (normal, uniform, exponential distributions, and Student’s t-distribution with 3, 5, and 7 degrees). Then, we applied MPTGA to the simulated data sets. The accuracy were greater than 99% for T3, T5, and exponential distributions and 100% for other distributions tested. The results are summarized in Fig. 8. In general, MPTGA was robust to residuals deviated from a normal distribution.

It is worth to note that there were lots of assumptions made in the MPTGA model, such as cubic polynomial function for mean trajectories and covariances related in first order auto correlation. The choices were made mainly due to limited number of time points in a time series data set. If a long time series is available, high degree polynomial functions and higher order correlation structure should be explored.

## Conclusions

Transcriptome response to virus infection may vary between viruses and over time. The Multivariate Polynomial Time-dependent Genetic Association (MPTGA) method can be applied to detect virus-specific effects on temporal gene expression traits. It is shown to enhance the power and specificity of detection subtype specific effects, which could result a more accurate target gene set for understanding the virus pathology.

## Methods

### MPTGA and extension

Lin et al. [6] proposed a Multivariate Polynomial Time-dependent Genetic Association (MPTGA) method to detect genetic effects in temporal gene expression trajectories. The MPTGA method assumes that for each genotype *j*, the temporal gene expression trait **y** across *m* time points follows a multivariate normal distribution $\mathcal {N}(\mathbf {g}_{j},\,\Sigma)$, with density function

$$f_{j}(\mathbf{y})=\frac{1}{(2\pi)^{m/2}|\Sigma|^{1/2}}\exp{\left[-\left(\mathbf{y}-\mathbf{g}_{j}\right)\Sigma^{-1}\left(\mathbf{y}-\mathbf{g}_{j}\right)^{T}/2\right]}. $$

The mean vector **g**_*j*_ for genotype *j* is modeled with a polynomial curve

$$\mathbf{g}_{j}=\left[\mathbf{g}_{j}(t)\right]_{1\times m}=\left[\Sigma_{k=0}^{K} \beta_{kj}t^{k}\right]_{1\times m},$$

where K is the degree of the polynomial function. In this study, K was set to be 3.

In particular, MPTGA assumes a first order auto-regression model to take into account the auto-correlation between different time points, with the covariance matrix specified as

$$\Sigma=\sigma_{e}^{2}\begin{bmatrix} 1 & \rho & \dots & \rho^{m-1} \\ \rho & 1 & \dots & \rho^{m-2} \\ \vdots & \vdots & \ddots & \vdots \\ \rho^{m-1} & \rho^{m-2} & \dots & 1 \end{bmatrix}. $$

For each sample *i*, its gene expression trajectory **y**_*i*_(*t*) across *m* time points can be written as

$$\mathbf{y}_{i}(t)=\delta_{i0}\sum_{k=0}^{K} \beta_{k0}t^{k} + \delta_{i1}\sum_{k=0}^{K} \beta_{k1}t^{k} +\epsilon_{i},$$

where *δ*_*i*0_ and *δ*_*i*1_ are the indicator variables of sample *i* taking genotype 0 or 1.

Given the joint likelihood

$$\mathcal{L}(\Theta)=\prod_{i=1}^{N}[\!\delta_{i0}f_{0}(\mathbf{y}_{i}) + \delta_{i1}f_{1}(\mathbf{y}_{i})]$$

The maximum likelihood estimates of the parameter set $\Theta =\left (\beta _{kj},\rho,\sigma _{e}^{2}\right)$ can be derived as described in the Lin et al. [6] paper.

Lin et al. [6] compared longitudinal trajectories between two different conditions (e.g. two possible genotypes in a haploid system). The method can be extended to accommodate three or more conditions (e.g. effects of three possible genotypes in a diploid system; e.g. effects of different viruses infection). This comparison can be done using done using a pairwise test between conditions. Alternatively, we can construct a full model to detect the (genetic) effects:

$$y_{i}(t)=\delta_{i0}\sum\limits_{k=0}^{K} \beta_{k0}t^{k} + \delta_{i1}\sum\limits_{k=0}^{K} \beta_{k1}t^{k} + \delta_{i2}\sum_{k=0}^{K} \beta_{k2}t^{k} + \epsilon_{i}$$

for a given trait Y, then the reduced model *H*_0_ (single gene expression trait curve)

$$H_{0}: \beta_{k0}=\beta_{k1}=\beta_{k2} \qquad \text{for all} k$$

can be compared against the full model *H*_1_ (different gene expression trait curve for different conditions (genotypes/viruses infection)):

$$H_{1}: \text{at least one of the equalities does not hold}$$

to test the hypothesis of the existence of eQTL at a locus or difference between effects of viruses by estimating these parameters with a MLE procedure and performing a likelihood ratio test. The full model approach differs from the pairwise test in that it imposes an extra condition of a shared covariance structure among all three conditions, which is estimated in the full model taking all three conditions into account; whereas the pairwise test only requires the covariance structure to be shared between each pair, which is estimated separately for each pair.

Similar to the MPTGA model with two genotypes, we assume that for each genotype *j*=0,1,2, the mean vector **g**_*j*_ for genotype *j* is modeled with a polynomial curve

$$\mathbf{g}_{j}=\left[\mathbf{g}_{j}(t)\right]_{1\times m}=\left[\Sigma_{k=0}^{K} \beta_{kj}t^{k}\right]_{1\times m}\qquad j=0,1,2.$$

Given the joint log-likelihood

$$\begin{array}{*{20}l} \log\mathcal{L}(\Theta)=&\sum\limits_{i=1}^{N}\delta_{i0}\log f_{0}(\mathbf{y}_{i}) +\sum\limits_{i=1}^{N} \delta_{i1}\log f_{1}(\mathbf{y}_{i}) \\ &+ \sum\limits_{i=1}^{N}\delta_{i2}\log f_{2}(\mathbf{y}_{i}), \end{array} $$

the maximum likelihood estimates of the parameter set $\Theta =\left (\beta _{0j},\beta _{1j},\beta _{2j},\beta _{3j},\rho,\sigma _{e}^{2}\right),j=0,1,2$ can be derived by first looking for the critical point of $\log \mathcal {L}(\Theta)$ by taking its derivative with respect to *β*’s and $\sigma _{e}^{2}$, finding that both $\beta 's,\sigma _{e}^{2}$ can be expressed as functions of *ρ* at the critical point, therefore $\log \mathcal {L}(\Theta)$ can be expressed as functions of *ρ* at the critical point as well; then the MLE of *ρ* can be derived by taking derivative of $\log \mathcal {L}(\Theta)$ with respect to *ρ*, and thus *β*’s and $\sigma _{e}^{2}$ can be obtained accordingly.

The detailed derivation is as follows. Define $\mathbf {T}_{j}=\sum _{i=1}^{N}\delta _{ij}\mathbf {y}_{i},j=0,1,2$; **I**_0_= [1⋯1]_1×*m*_,**I**_1_= [1⋯*m*], $\mathbf {I}_{2}=\mathbf {I}_{1.}^{2}=\ [1\cdots m^{2}]$, $\mathbf {I}_{3}=\mathbf {I}_{1.}^{3}=\ [1\cdots m^{3}]$; $Q(\rho,\mathbf {U},\mathbf {V})=\frac {1}{1-\rho ^{2}}(U_{1}V_{1}+U_{m}V_{m})-\frac {\rho }{1-\rho ^{2}}\left [\sum _{i=1}^{m-1}U_{i}V_{i+1}+U_{i+1}V_{i}\right ]+\frac {1+\rho ^{2}}{1-\rho ^{2}}\sum _{i=2}^{m-1}U_{i}V_{i}$, where **U**= [*U*_1_,⋯,*U*_*m*_] and **V**= [*V*_1_,⋯,*V*_*m*_].

Taking derivative of $\log \mathcal {L}(\Theta)$ with respect to *β*_.0_’s gives the following linear system:

$$\begin{bmatrix} \alpha_{11}\beta_{00}+\alpha_{12}\beta_{10} + \alpha_{13}\beta_{20} + \alpha_{14}\beta_{30}=b_{1} \\ \alpha_{21}\beta_{00}+\alpha_{22}\beta_{10} + \alpha_{23}\beta_{20} + \alpha_{24}\beta_{30}=b_{2} \\ \alpha_{31}\beta_{00}+\alpha_{32}\beta_{10} + \alpha_{33}\beta_{20} + \alpha_{34}\beta_{30}=b_{3} \\ \alpha_{41}\beta_{00}+\alpha_{42}\beta_{10} + \alpha_{43}\beta_{20} + \alpha_{44}\beta_{30}=b_{4} \end{bmatrix},$$

where *α*_*ij*_=*n*_0_*Q*(*ρ*,**I**_*i*−1_,**I**_*j*−1_), *b*_*i*_=*Q*(*ρ*,**T**_0_,**I**_*i*−1_), $n_{0}=\sum _{i=1}^{N}\delta _{i0}$. Then *β*_.0_’s can be estimated by

$$\begin{bmatrix} \beta_{00}\\ \beta_{10}\\ \beta_{20}\\ \beta_{30} \end{bmatrix}=\begin{bmatrix} \alpha_{11}&\alpha_{12}& \alpha_{13}& \alpha_{14} \\ \alpha_{21}&\alpha_{22}& \alpha_{23}& \alpha_{24}\\ \alpha_{31}&\alpha_{32}&\alpha_{33}&\alpha_{34}\\ \alpha_{41}&\alpha_{42}&\alpha_{43}&\alpha_{44} \end{bmatrix}^{-1}*\begin{bmatrix} b_{1}\\ b_{2}\\ b_{3}\\ b_{4} \end{bmatrix}.$$

Similarly, define $n_{1}=\sum _{i=1}^{N}\delta _{i1}$, $\alpha _{ij}^{(1)}=n_{1}Q(\rho,\mathbf {I}_{i-1},\mathbf {I}_{j-1})$, $b_{i}^{(1)}=Q(\rho,\mathbf {T}_{1},\mathbf {I}_{i-1})$; $n_{2}=\sum _{i=1}^{N}\delta _{i2}$, $\alpha _{ij}^{(2)}=n_{2}Q(\rho,\mathbf {I}_{i-1},\mathbf {I}_{j-1})$, $b_{i}^{(2)}=Q(\rho,\mathbf {T}_{2},\mathbf {I}_{i-1})$; then *β*_._’s can be derived accordingly.

Next, taking derivative with respect to $\sigma _{e}^{2}$ gives

$$\begin{array}{*{20}l} \sigma_{e}^{2}=&\left\{\sum_{i=1}^{N}Q(\rho,\mathbf{y}_{i},\mathbf{y}_{i})+\sum_{i=0}^{2}\left[Q(\rho,\mathbf{T}_{i},\mathbf{T}_{i})\right.\right.\\ &-\sum_{j=0}^{3} 2\beta_{ji}Q\left(\rho,\mathbf{T}_{i},\mathbf{I}_{j}\right)\\ &\left.+\sum_{j=0}^{3}\sum_{k=0}^{3} n_{i}\beta_{ji}\beta_{ki}Q(\rho,\mathbf{I}_{j},\mathbf{I}_{k})]\right\}/(mN) \end{array} $$

With the previously derived estimators of *β*’s and $\sigma _{e}^{2}$ also as functions of *ρ*, the log-likelihood can now be written as

$$\begin{array}{*{20}l} \log\mathcal{L}(\Theta)=&-\frac{mN}{2}\log 2\pi-\frac{N}{2}\left[(m-1)\log\left(1-\rho^{2}\right)\right.\\ &\left.+m\log\sigma_{e}^{2}\right]-\frac{mN}{2} \end{array} $$

Based on the MLE of the unknown parameters, a LRT $\left (-2\log \Lambda \sim \chi _{8}^{2}\right)$ can be conducted comparing the aforementioned full model *H*_1_ with the null model *H*_0_ to test for virus-specific effects.

### Detecting virus-specific effects on post-infection temporal expression

The MPTGA method can be applied to a broader setting that compares the temporal gene expression traits between two different conditions, whereas the original setting proposed by Lin et al. [6] is a special case that compares two possible genotypes in a haploid system. In this paper, we are interested in detecting the virus-specific effects on the post-infection temporal gene expression trajectories. For a given virus *v*, we define *j*=0,1 as the indicator variable of a sample infected by virus *v* or other viruses. The null hypothesis *H*_0_ assumes that the temporal gene expression trait **y**_*j*_ share the same trajectory pattern after infected by virus *v* and other viruses:

$$H_{0}: \beta_{k0}=\beta_{k1} \qquad \text{for all}\ k$$

which is tested against the full model *H*_1_, assuming that the temporal post-infection gene expression trait **y**_*j*_ with virus *v* has a unique trajectory pattern distinct from the post-infection trajectories with other viruses:

$$H_{1}: \text{at least one of the equalities does not hold}$$

The hypothesis can be tested using a likelihood ratio test.

Similarly, MPTGA can be applied to cases with three or more groups as outlined above.

## Abbreviations

ENCODE: Encyclopedia of DNA elements
GEO: Gene expression omnibus
GO: Gene ontology
ILC: Innate lymphoid cell
LRT: Likelihood ratio test
MLE: Maximum likelihood estimation
MPTGA: Multivariate polynomial time-dependent genetic association
NK: Natural killer
